# An investigation of excess residual cytoplasm in human spermatozoa and its distinction from the cytoplasmic droplet

**DOI:** 10.1186/1477-7827-10-92

**Published:** 2012-11-17

**Authors:** Anil K Rengan, Ashok Agarwal, Michelle van der Linde, Stefan S du Plessis

**Affiliations:** 1 Center for Reproductive Medicine, Glickman Urological and Kidney Institute, Cleveland Clinic Foundation, Cleveland, Ohio, 44195, USA; 2 Medical Physiology, Faculty of Medicine and Health Sciences, Stellenbosch University, Francie van Zijl Avenue, Tygerberg, 7507, South Africa

**Keywords:** Cytoplasmic droplet, Excess residual cytoplasm, Midpiece, Spermiogenesis, Cytoplasmic extrusion, Residual body, Regulatory volume decrease, Reactive oxygen species, Oxidative stress, Peroxidative damage

## Abstract

Recent studies have shown cytoplasmic droplets to be normal morphological occurrences in human male spermatozoa. When the cytoplasm around the sperm midpiece is present in large amounts, however, pathological effects may transpire. The cytoplasmic droplet then becomes known as excess residual cytoplasm, which can impair overall sperm function and produce higher levels of reactive oxygen species, potentially leading to male infertility. Though the distinction between cytoplasmic droplets and excess residual cytoplasm has been made, some studies fail to recognize the difference and incorrectly label the latter as a cytoplasmic droplet. This review attempts to clarify excess residual cytoplasm’s effect on fertility, examine the enzymes responsible, and suggest tests and possible treatment options for those affected by this defect.

## Background

Male infertility accounts for about half of all infertility cases and may arise from a variety of factors. One known cause is the retention of excess cytoplasm around the midpiece due to an arrest in spermiogenesis and incomplete cytoplasmic extrusion [1]. This is now known as excess residual cytoplasm (ERC). In comparison to the typical cytoplasmic droplet (CD) found in ejaculated human spermatozoa, ERC contains elevated levels of cytoplasm enzymes that produce pathological amounts of reactive oxygen species (ROS) [2]. The high ROS levels may then result in oxidative stress (OS). ERC ultimately affects sperm motility [3], morphology [2] and fertilization potential [4], thereby leading to male infertility. The purpose of this review is to compare CDs to ERC, to describe ERC’s relevance to human reproduction, and to clarify its assessment and clinical importance.

## Review

### The cytoplasmic droplet

Retzius first identified the normal CD in 1909, the significance of which has eluded scientists ever since [5]. It is a familiar element of mammalian spermatozoa, though many studies in the past have focused primarily on CDs in non-human species. The differences between human and non-human mammals became apparent once researchers drew attention to human CDs. Unlike those of domestic species (i.e. boars, rams, goats, etc.), CDs of normal human spermatozoa are still present after ejaculation [6]. For this reason, CDs are not considered detrimental to proper sperm function.

### Structure

**Figure 1:**
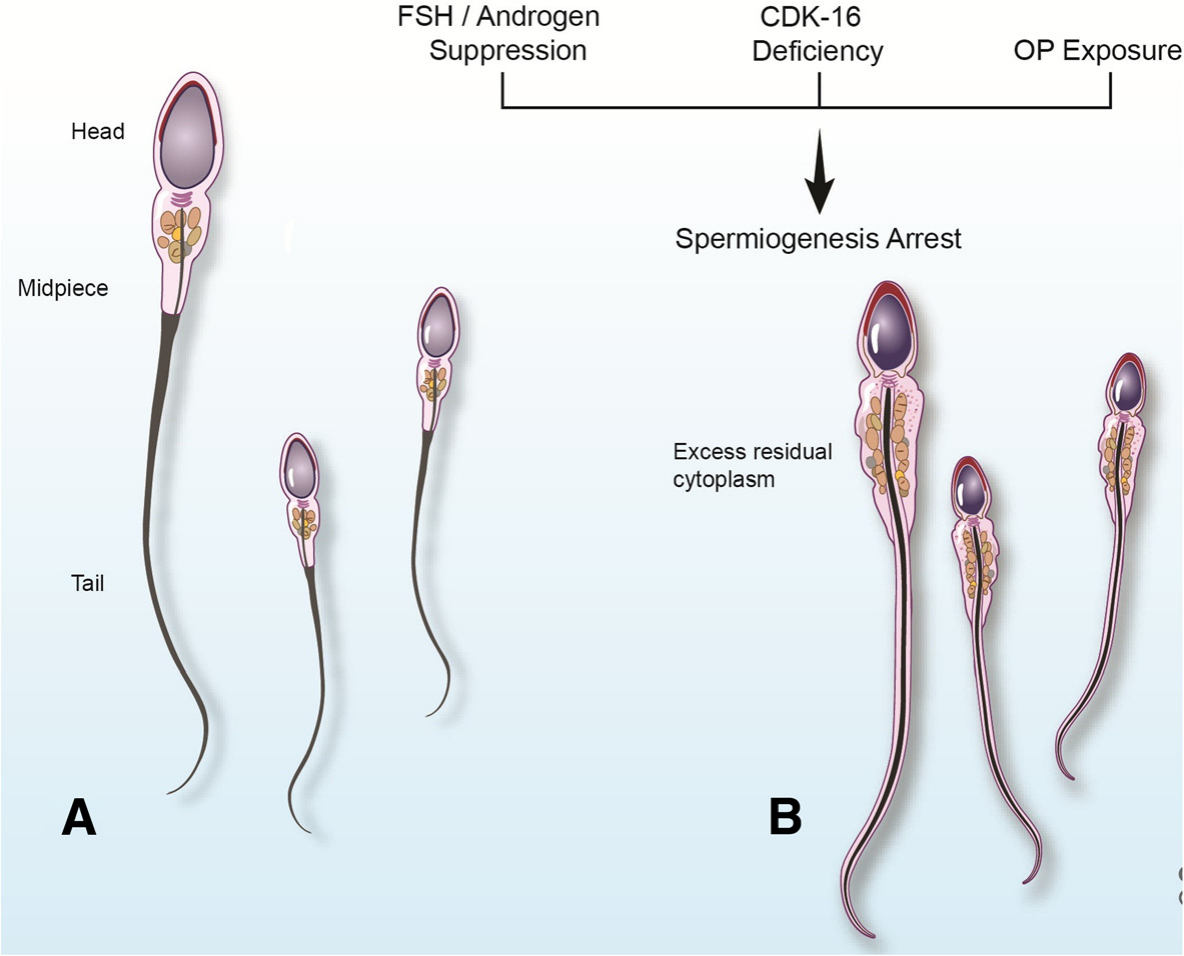
**CD vs. ERC Structure.** Illustration comparing (**A**) spermatozoa with typical cytoplasmic droplets (CD) and (**B**) spermatozoa presenting with excess residual cytoplasm (ERC). Also listed are specific causes of spermiogenesis arrest that can lead to ERC.

Mammalian CDs are surrounded by a cell membrane and contain cytoplasm that houses the cytosol and cytoskeletal network [7]. Hermo and coworkers [8] conducted studies on rat CDs, and in these species, the droplet comprises of lamellae and small vesicles unlike structural components of the Golgi apparatus and endoplasmic reticulum. The CDs of mature non-human mammalian sperm are found at the distal end of the midpiece. Mature human spermatozoa are similar to that of other mammals in possessing a CD at the midpiece. However, the human CD is more proximal, located at the neck as opposed to the end of the annulus [9] (Figure 1A). Due to the mitochondrial helix that forms around the core of the axoneme-outer dense fibers complex, the midpiece has a large diameter relative to the rest of the cell [10].

The mammalian CD is approximately 2 μm in diameter [11] and is comprised of lipids, lipoproteins, RNAs, and a variety of hydrolytic enzymes [12]. It also contains ion channels and golgi-derived vesicles [10].

The midpieces of human spermatozoa contain osmotically sensitive “midpiece vesicles” (MPVs) as confirmed by Cooper and colleagues [9]. MPVs were previously considered distinct from CDs and characteristic of immature sperm [13]. However, Cooper and colleagues suggested that MPVs and CDs are one and the same as neither withstands air-drying well.

### Manifestation

As a result of spermatogenesis and epididymal maturation, male germ cells differentiate to become fully functional spermatozoa. Spermiogenesis, the final stage of spermatogenesis, transforms haploid spermatids to free testicular (non-motile) spermatozoa [14]. It is during the last (“maturation”) phase of spermiogenesis that the CD is formed [15].

This is accomplished through the actions of Sertoli cells by a process known as “cytoplasmic extrusion” [16], which occurs before sperm are transported to the epididymis [17]. Cytoplasmic extrusion, along with various other maturation steps, is essential for allowing zona binding capacity and fertilization potential of spermatozoa [16].

In the tubular lumen of the testis, Sertoli cells extrude and phagocytose most of the germ cell cytoplasm as “residual bodies”, the remnant of which becomes the CD [17]. In most non-human mammalian species, the droplet migrates down the tail and is finally shed around the time of ejaculation [6]. However, the exact mechanism of this movement has not yet been clarified [5]. Retention of CDs on these non-human ejaculated spermatozoa has been associated with infertility [18,19]. Human spermatozoa, however, normally retain a small amount of the droplet around the midpiece after spermiogenesis.

### Physiology

Because they possess only a small amount of cytoplasm, spermatozoa lack the organelles required for osmoregulation [20]. Therefore, spermatozoa rely on their extracellular environment for osmoregulation. The midpiece is an ideal location for the CD; this is the major site of water influx and cell volume regulation, which is important when spermatozoa face hypo-osmotic challenges (e.g. cervical mucus osmolality) [21].

Regulatory volume decrease (RVD) has been shown to occur in spermatozoa under hypotonic conditions [22], which compensates for any swelling during and after ejaculation [6]. This occurs because CDs contain osmolyte channels that facilitate osmosis. K^+^ and Cl^-^ channels have also been found at the neck and along the midpiece [22].

The epididymis is responsible for osmolyte loading via regulatory volume increase (RVI) [12]. The amount of osmolytes present is vital for proper sperm function; if adequate, motility will be “forward progressive” for successful transit through the female reproductive tract [6]. In the case of low osmolyte loading or presence of cryoprotectants or an osmolyte channel blocker (e.g. quinine), spermatozoa swell and forward progression is thwarted [23,24].

Droplets in non-human mammalian spermatozoa can induce swelling and flagellar angulation [25], which inhibit progressive motility and are associated with infertility. Human spermatozoa, on the other hand, retain their CDs with no negative effects, because there is no angulation or coiling effect on the flagellum under naturally hypotonic conditions [6].

Chen and coworkers [26] have reported that aquaporin 3 (AQP3) is a water channel in human spermatozoa necessary for RVD. Their research suggests that AQP3 balances reduction in sperm motility due to any cell swelling that may occur.

The CD is home to a variety of enzymes and receptors. Köhn and colleagues [27] identified angiotensin-converting enzyme (ACE) to be present in the CD, a membrane-bound enzyme that is released during capacitation. In this study, it was shown that less ACE is present among more motile spermatozoa, signifying a negative correlation between ACE level and sperm maturity.

15-lipoxygenase (15-LOX) and components of the ubiquitin-dependent proteolytic pathway are prominent in the CDs of mammalian spermatozoa [28]. 15-LOX may be responsible for the removal of CDs from spermatozoa and are thought to participate in epididymal sperm maturation and formation of the midpiece and mitochondrial sheath. The components of the ubiquitin-proteasome pathway are believed to assist in spermiogenesis and organelle degradation. Though this study was performed on boar spermatozoa, 15-LOX and ubiquitin components were detected in human spermatozoa as well.

Calreticulin (CRT) and the inositol 1,4,5-trisphosphate receptor (IP3R) have also been found within vesicles of the CD [29]. Both are implicated in calcium level oscillations during hyperactivation and the acrosome reaction. Ropporin is yet another protein found in both the CD and flagellum that is suggested to have a role in regulating sperm motility and the acrosome reaction by binding to the amphipathic helix region of A-kinase anchoring proteins (AKAPs) [30].

Miranda-Vizuete and colleagues [31] have reported the localization of sperm-specific thioredoxin (Sptrx) in the CD of human spermatozoa. Sptrx was shown to behave as a reductant, possibly to correct wrong disulfide pairings during sperm tail formation. Failure in Sptrx expression has been linked to dysplasia of the fibrous sheath (DFS) [31], or “stump tail syndrome” [32], which can cause severe asthenozoospermia or sperm immotility.

Among the assortment of CD enzymes are creatine kinase (CK), lactic acid dehydrogenase (LDH), superoxide dismutase (SOD), and glucose-6-phosphate dehydrogenase (G6PDH) [15], all of which are involved in the energy metabolism of the CD. Defective CDs harbor higher levels of these metabolic enzymes, which can impair overall sperm function.

**Figure 2:**
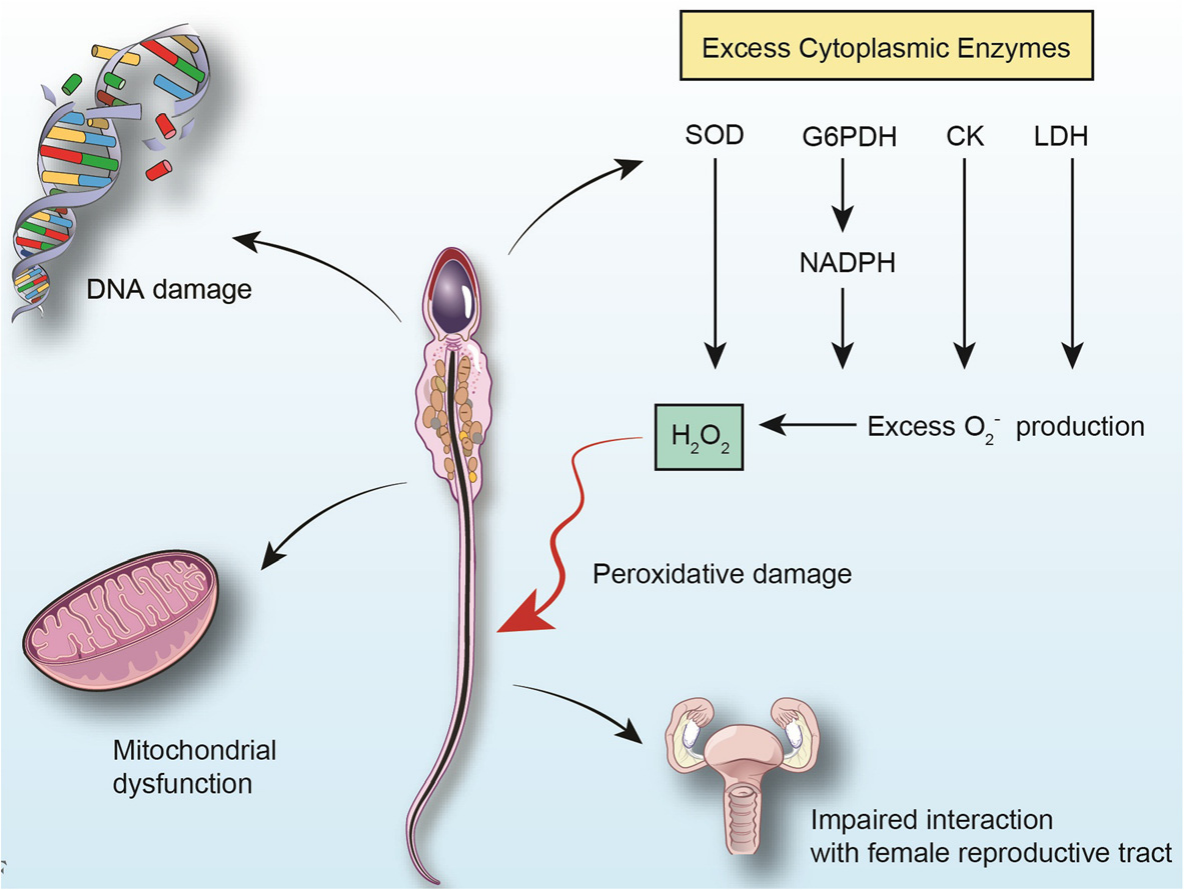
**Problems Associated with ERC.** Illustration detailing the pathological effects of excess residual cytoplasm (ERC), including peroxidative damage to the sperm membrane, DNA damage, mitochondrial dysfunction, and impaired sperm function within the female reproductive tract.

The World Health Organization (WHO) considers CDs to be defects when they are larger than one-third of the sperm head size [33] (Table 1). This ERC arises due to the premature arrest of spermiogenesis [9]. Unlike most mammalian species, human spermatozoa are unable to modify any residual cytoplasm that may exist during epididymal maturation or at ejaculation [34]. Compared to the typical CD, ERC possesses a surplus of the aforementioned enzymes, which can negatively affect sperm function and lead to male infertility (Figure 2).

**Table 1:** Complete CD and ERC comparison

	**Cytoplasmic droplet**	**Excess residual cytoplasm**
*Location*	Proximal part (neck) of midpiece	Along midpiece
*Structure*	Cytoplasm smaller than 1/3 sperm head size, 2 μm in diameter	Cytoplasm larger than 1/3 to 1/2 sperm head size
*Caused by*	Cytoplasmic extrusion via Sertoli cell phagocytosis	Spermiogenesis arrest and interruption of cytoplasmic extrusion
*Function/Consequences*	Physiological – Regulatory volume decrease and ROS production	Physiological – Elevated levels of cytoplasm enzymes; Pathological – Incomplete maturation (“dysmature”), oxidative stress, lipid peroxidation, and apoptosis
*Identification/Testing Methods*	Air-drying (does not survive) and binary image analysis via NADH/NBT staining	Air-drying (survives), binary image analysis via NADH/NBT staining, immunofluorescence, immunoblotting, ROS markers, aniline blue chromatin staining, and Sptrx screening

Differences between cytoplasmic droplets (CDs) and excess residual cytoplasm (ERC) with regards to location, structure, cause, function/consequences, and identification/testing methods, and hallmarks.

### Excess residual cytoplasm

Until recently, the concept of ERC was not as widely accepted throughout the scientific community. Instead, a variety of words were used indiscriminately with regards to both normal and abnormal cytoplasmic residue [5]. This discrepancy was settled in a study conducted by Cooper and colleagues [9] that helped bring this distinction to the forefront. Their research demonstrated a major difference; ERC survives the air-drying techniques used for human seminal smears whereas CDs do not. ERC may span the entire midpiece area although the human CD is found at the neck of the midpiece (Table 1).

Since ERC-bearing spermatozoa are unable to complete maturation [5]**,** some may consider sperm bearing ERC as “immature”. However, the term “dysmature” is deemed more appropriate [35]. “Immature” is mostly used to describe normal spermatozoa that have yet to undergo epididymal maturation after release from the testis. “Dysmature”, on the other hand, describes spermatozoa that have experienced an interruption or cessation of spermiogenesis and/or epididymal maturation.

### Manifestation

Spermatozoa retain excess cytoplasm due to a disruption of spermiogenesis as evidenced by high levels of cytoplasmic enzymes [2] (Table 1). This process has yet to be detailed, but there are various explanations as to how this may occur.

For example, suppression of FSH and/or androgens (primary hormone regulators of spermatogenesis) can cause spermiogenesis arrest [36] (Figure 1B). D’Souza and coworkers [37] demonstrated that administration of exogenous 17-beta-estradiol led to reduced FSH and intratesticular testosterone levels, thereby leading to an absence of tubulobulbar complexes.

The apically located tubulobulbar complex is an actin-based structure that anchors late spermatids to the Sertoli cell and indirectly produces the residual body during cytoplasmic extrusion [38]. During formation of the tubulobulbar complex, there is a considerable loss of cytoplasm in the spermatid, implicating these complexes in Sertoli cell phagocytosis [39]. Disruption of tubulobulbar complex formation is therefore associated with retention of excess cytoplasm around the midpiece.

Cyclin-dependent kinase 16 (CDK16), which is highly expressed in the brain and testis, has also been associated with spermiogenesis. Mikolcevic and coworkers [40] determined that CDK16 deficiency in mice is correlated with morphologically defective spermatozoa possessing malformed heads and ERC (Figure 1B). The precise role of CDK16 in spermatozoa maturation is still unclear.

Recently, there have been reports associating organophosphorus pesticide (OP) exposure and reduced sperm function in people and laboratory animals. OPs, such as dichlorvos (DDVP), are used as pesticides to shield crops and homes from insect attack. A study conducted by Okamura and colleagues [41] revealed a higher incidence of cytoplasm in rat spermatozoa with DDVP exposure (Figure 1B). Malathion, another OP, has been shown to increase cytoplasm in rat spermatozoa. Researchers suggest that such pesticides trigger an early arrest of spermatozoa maturation, which may be alleviated by reducing OP exposure.

Studies have suggested the association of ERC with varicocele presence [42]. They each cause an increase in ROS production, and both idiopathic and varicocele-related male infertility have been correlated with impaired cytoplasmic extrusion [43]. Infertile men with varicocele were shown to have the highest percentage of sperm presenting with ERC. The mechanism by which varicocele and ERC are related has yet to be elucidated.

Smoking has also been shown to impair cytoplasmic extrusion and sperm function [44]. The exact pathophysiology is uncertain, but smoking may have effects on Sertoli and/or Leydig cell function [45] and/or oxidative balance in the testis [46]. Since smoking may be associated with a lifestyle of other poor health decisions, such as alcohol/drug abuse and suboptimal diet, confounding factors may further affect a couple’s fertility [44].

### Pathology

ERC has many health implications spanning a wide range of disorders (Table 1). Problems primarily arise due to the elevated levels of key enzymes found within the cytoplasm itself (Figure 2).

Normally, spermatozoa produce a low level of ROS from their mitochondria [35]. Physiological levels of ROS trigger and modulate tyrosine phosphorylation, for instance, which elicits vital functions like capacitation and the acrosome reaction [34].

When ROS levels are raised, as is the case with ERC, spermatozoa are limited in their ability to eradicate the surplus. This is typically due to the presence of extra electron transport chains in the plasma membrane, unfamiliar oxidases, or oxidoreductases that promote xenobiotic production [34]. Physiological levels of antioxidants, in its limited availability, cannot counteract this excess production of ROS. In fact, electron leakage from the sperm mitochondrial electron transport chain is considered to be a major source of ROS generation in defective sperm [47]. Sperm motility has been shown to decrease as mitochondrial production of ROS increases, subsequently inducing DNA damage.

As a result of high ROS levels, spermatozoa may assume a state of oxidative stress, characterized by damage to both mitochondrial and nuclear DNA [48] along with peroxidative damage to the sperm plasma membrane [49]. It is presumed that peroxidative damage is associated with higher activities of CK and G6PDH [50]. The ROS not only affects the abnormal spermatozoa that generates it, but it can also damage normal spermatozoa as well [51].

In terms of energy metabolism, ERC contains higher levels of G6PDH, resulting in greater NADPH production. By means of the hexose monophosphate shunt, G6PDH produces NADPH and controls the levels of glucose flux and NADPH availability [15]. Spermatozoa use this shunt for its supply of electrons for ROS generation. NADPH is a substrate for ROS-generating NADPH oxidases [15]. The superoxide anion, O_2_ **·** ^-^, is one common ROS that is converted to H_2_O_2_ via SOD, another enzyme commonly found in ERC [52]. H_2_O_2_ molecules, if left to accumulate, may then undergo homolytic cleavage to form two hydroxyl free radicals (OH **·** ^-^), highly reactive electrophiles that cause oxidative damage to cells [53] (Figure 2). Due to their high phospholipid content and relatively low cytoplasmic volume, spermatozoa are especially susceptible to this condition [34]. LDH, also involved in the maintenance of spermatozoa energy metabolism, has not been shown to be directly injurious to the cells in higher levels [54].

Previous studies have indicated that sperm mitochondria release cytochrome C in response to ROS stimulation [55], which activates a signaling cascade involving caspase 3 and 9 that ultimately leads to sperm apoptosis. Elevated ROS levels have been associated with increased instances of apoptosis [56]. Because caspase 3 activation is localized to the midpiece, Weng and coworkers [57] have suggested that apoptotic mechanisms may stem from the midpiece cytoplasm and then operate in the nucleus.

Under physiological conditions, normal ROS levels have been correlated with reduced DNA damage via chromatin cross-linking [58]. When ROS levels increase, the subsequent oxidative stress induces DNA damage [59]. DNA repair in spermatozoa and the ability of sperm to undergo apoptosis deteriorates during late spermatogenesis [15]. Both mitochondrial and nuclear DNA can be affected, the former especially susceptible due to insufficient protection against ROS attack [35]. This may not pose as great a risk since sperm mitochondria are discarded in the oocyte following fertilization [60]. However, any damaged nuclear DNA will be incorporated into the zygote. Even if peroxidative membrane damage occurs, damaged DNA can still be transferred into the embryo [61]. Therefore, the oocyte is responsible for correcting any DNA damage or inducing apoptosis (i.e. embryonic loss) before the first cleavage. Any lingering errors following these preventative measures may affect the eventual development and overall health of the offspring.

ERC can also affect how a spermatozoon functions within the female reproductive system. Oxidative stress caused by ERC can impair sperm motility and function, affecting capacitation and fertilization [62]. Furthermore, it may render the sperm plasma membrane unable to respond to intracellular calcium signals, impeding sperm-oocyte fusion [63]. This may involve reduced plasma membrane fluidity [64] in addition to changes to membrane-bound enzyme activity (e.g. ion channels) [65]. Defective enzyme expression may affect fertilization in that an abundance of 15-LOX in semen can cause early acrosomal exocytosis [66], possibly hampering zona binding and penetration [67]. Oxidative stress in human spermatozoa impairs both fertilization potential [68] and embryonic/fetal development [69]. It can lead to higher instances of miscarriage [70], and offspring morbidity, including childhood cancer [71].

### Identification

**Figure 3:**
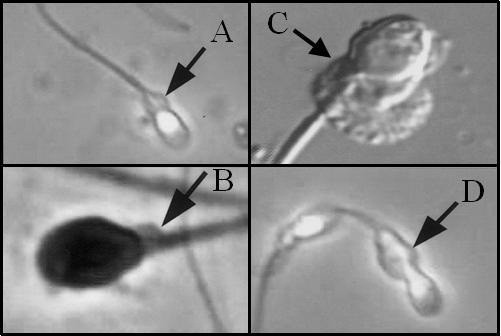
**CD and ERC under Microscope.** True examples of cytoplasmic droplets (**A**, **B**) and excess residual cytoplasm (**C**, **D**) in human spermatozoa as examined by microscopy. This image was modified from Cooper and colleagues [9] with permission, © Oxford Journals.

There are a variety of known methods for ERC analysis (Table 1). Microscopy can be used to estimate the size of the cytoplasm around a sperm head (Figure 3). CDs larger than one-third of the sperm head size are classified as ERC [33].

An image analysis for ERC has been developed via midpiece staining to generate binary images [2]. The staining, which renders the entire midpiece blue-black, uses NADH and nitroblue tetrazolium (NBT) as an electron donor and acceptor, respectively. Once the binary images are created, morphometric analysis is performed in order to gauge the extent of excess cytoplasm retention. Though this technique is more time-consuming than biochemical assays, it provides information on a cellular level that may be used as selection criteria for intracytoplasmic sperm injection (ICSI) during assisted reproduction.

Rago and colleagues [1] have demonstrated the presence of two estrogen receptors within ERC, ER-alpha and ER-beta. The presence of the ER-beta form in abnormal sperm and its absence in normal spermatozoa suggest a role for estrogens in sperm maturation. These receptors can be monitored using immunofluorescence and immunoblotting techniques.

The defenselessness that sperm mitochondrial DNA exhibits under ROS attack makes it a sensitive marker for examining oxidative stress [35] as the DNA damage can be accessed through the TUNEL assay, for example. To assess the extent of DNA damage, further screening (e.g. aniline blue chromatin staining, which tests for histone integrity) should be performed [72].

Screening of Sptrx status in ERC-bearing spermatozoa may provide useful information to clinicians, in particular, on sperm tail pathologies [31]. Therefore, Sptrx screening may facilitate the diagnosis of patients affected with DFS.

### Interventions and treatments

CK, which is sperm-specific and expressed in the testis, is a marker of cell maturity. Expression of the CK-M isoform, also known as HspA2 [73], is associated with changes in the sperm plasma membrane as well as cytoplasmic extrusion [16]. CK levels can be tested with immunochemistry while CK-M/HspA2 antiserum is used to indicate maturity by highlighting the ERC [74].

In order to manage oxidative stress, a variety of antioxidant treatments exist to reduce ROS levels by becoming radicals themselves [69]. This therapy could be used on patients presenting with a high OS status [72]. Glutathione and selenoproteins are example of such antioxidants. alpha-Tocopherol, a form of vitamin E [75], and vitamin C may be used together *in vivo* to prevent peroxidative damage [76]. A cofactor of the pyruvate dehydrogenase complex, alpha-lipoic acid, has also been shown to significantly reduce reactive oxygen metabolites *in vivo*[77]. Ascorbate and catalase have been shown to reduce ROS (specifically H_2_O_2_) *in vitro* as well [78].

Because ERC and subsequent peroxidative damage to the sperm plasma membrane can compromise sperm-oocyte fusion, ICSI can be used as an intervention to bypass this obstacle. Nevertheless, other defects may still persist, such as nuclear DNA damage, preventing proper embryonic/fetal development [71].

Moreover, varicocelectomy has been shown to improve cytoplasmic extrusion and reduce excess ROS production in men with ERC [42]. This topic merits further investigation to assess the efficacy of this and other treatments.

## Conclusions

The CD is a morphological feature unique to human spermatozoa and absent in other non-human mammals. It has functional significance and is involved in hyperactivation, capacitation, and the acrosome reaction. An abnormally large amount of cytoplasm around the sperm midpiece has pathological implications and is then termed “ERC” based on size and function. Having distinguished CDs from ERC, future studies should attempt to measure the incidence rate of ERC in human males and explain why human spermatozoa tend to retain CDs after maturation. Additionally, the pathophysiology of ERC must be described in greater detail with regards to male infertility. Furthermore, prospective studies should evaluate the efficacy of the various treatments for ERC. There is a lot to be learned about this particular morphological defect. Clarification is vital for better assessing male infertility in a clinical setting.

## Abbreviations

ERC: Excess residual cytoplasm; CD: Cytoplasmic droplet; ROS: Reactive oxygen species; OS: Oxidative stress; MPV: Midpiece vesicle; RVD: Regulatory volume decrease; RVI: Regulatory volume increase; AQP3: Aquaporin 3; ACE: Angiotensin-converting enzyme; 15-LOX: 15-lipoxygenase; CRT: Calreticulin; IP3R: ; AKAP: A-kinase anchoring protein; : Inositol 1,4,5-trisphosphate receptor; Sptrx: Sperm-specific thioredoxin; DFS: Dysplasia of the fibrous sheath; CK: Creatine kinase; LDH: Lactate acid dehydrogenase; SOD: Superoxide dismutase; G6PDH: Glucose-6-phosphate dehydrogenase; WHO: World health organization; CDK16: Cyclin-dependent kinase 16; OP: Organophosphorus pesticide; DDVP: Dichlorvos; NBT: Nitroblue tetrazolium; ICSI: Intracytoplasmic sperm injection.

## Competing interests

The authors declare that they have no competing interests.

## Authors' contributions

AKR studied this topic and drafted this manuscript. AA served as a mentor and reviewer of this article. MVDL revised this manuscript. SSDP conceived of the review, participated in its design and coordination, and helped to draft the manuscript. All authors read and approved the final manuscript.
